# Influence of Waist-to-Hip Ratio on the Prognosis of Heart Failure Patients With Revascularized Coronary Heart Disease

**DOI:** 10.3389/fcvm.2021.732200

**Published:** 2021-10-01

**Authors:** Yingyue Zhang, Yan Zhang, Yajun Shi, Wei Dong, Yang Mu, Jing Wang, Yifan Gao, Rong Hu, Yong Xu, Yundai Chen, Jing Ma

**Affiliations:** ^1^Department of Cardiology, Chinese PLA General Hospital, Beijing, China; ^2^Medical School of Chinese PLA, Beijing, China; ^3^The First Affiliated Hospital of Dalian Medical University, Liaoning, China

**Keywords:** heart failure, coronary heart disease, waist-to-hip ratio, abdominal obesity, forecasting

## Abstract

**Background:** Heart failure (HF) is considered one of the most common complications of coronary heart disease (CHD), with a higher incidence of readmission and mortality. Thus, exploring the risk factors related to the prognosis is necessary. Moreover, the effect of the waist-to-hip ratio (WHR) on HF patients with revascularized CHD is still unclear. Thus, we aimed to assess the influence of WHR on the prognosis of HF patients with revascularized CHD.

**Methods:** We collected data of HF patients with revascularized CHD who were referred to the Cardiac Rehabilitation Clinic of PLA Hospital from June 30, 2015, to June 30, 2019. Cox proportional hazard regression analysis was used to determine the relationship between WHR and prognosis of HF patients with revascularized CHD. Patients were divided into higher and lower WHR groups based on the cutoff WHR value calculated by the X-tile software. Cox regression analysis was used to analysis the two groups. We drew the receiver operating characteristic curve (ROC) of WHR and analyzed the differences between the two groups. Endpoints were defined as major adverse cardiac events (MACE) (including all-cause mortality, non-fatal myocardial infarction, unscheduled revascularization, and stroke).

**Results:** During the median follow-up of 39 months and maximum follow-up of 54 months, 109 patients were enrolled, of which 91.7% were males, and the mean age was 56.0 ± 10.4 years. WHR was associated with the incidence of MACE in the Cox regression analysis (*p* = 0.001); an increase in WHR of 0.01 unit had a hazard ratio (HR) of 1.134 (95%CI: 1.057–1.216). The WHR cutoff value was 0.93. Patients in the higher WHR group had a significantly higher risk of MACE than those in the lower WHR group (HR = 7.037, 95%CI: 1.758–28.168). The ROC area under the curve was 0.733 at 4 years. Patients in the higher WHR group had a higher body mass index (BMI; 26.7 ± 3.5 vs. 25.4 ± 2.4, *P* = 0.033) than patients in the lower WHR group.

**Conclusions:** WHR is an independent risk factor of the long-term prognosis of Chinese HF patients with revascularized CHD. Patients with WHR ≥ 0.93 require intensified treatment. Higher WHR is related to higher BMI and ΔVO2/ΔWR.

## Introduction

The incidence rate of heart failure (HF) ranges from 1 to 2% of the population in a developed country. However, its incidence exceeds 10% in people aged over 70 (1, 2). Recently, the distribution of the etiology of HF in developed and developing countries gradually became similar: coronary heart disease (CHD) becomes the leading cause of HF (3, 4). One Chinese survey of 42 regions and over 10 thousand hospitalized HF patients showed that CHD accounted for 56% of the cause of HF (5). The progress in the treatment of CHD, such as revascularization techniques and optimal medical therapy, has reduced the mortality rate, consequently it also increased the number of HF patients with CHD (6, 7). This subgroup of patients usually brings a significant burden to social and medical insurance because of the high incidence of rehospitalization and mortality; however, strong evidence from diagnosis to treatment is still lacking (6, 8, 9). Therefore, finding the prognostic factors of HF patients with revascularized CHD is an urgent issue.

Studies reported a strong association between abdominal obesity and cardiac metabolic characteristics (10, 11). Moreover, abdominal obesity is established as one of the risk factors of CHD (12, 13). However, the effect of abdominal obesity on the prognosis of HF is still controversial. Some researchers pointed out that abdominal obesity was a risk factor of HF and is related to the increase of all-cause mortality (14, 15). Other researchers proposed that abdominal obesity was a “protective” factor of HF and related to the improvement of HF prognosis (16, 17). Surprisingly, little attention has been devoted to the influence of abdominal obesity on the prognosis of HF patients with CHD.

Abdominal obesity is measured by different methods such as computerized tomography (CT), magnetic resonance imaging (MRI), anthropometry measurements, and other bioelectrical impedance analysis. Anthropometry measurements includes waist circumference (WC), waist-to-height ratio (WHtR), and waist-to-hip ratio (WHR) (18). CT, MRI, and bioelectrical impedance analysis are the methods using direct and precise measurements of abdominal fat, but they are not widely used in the clinical work due to the high cost or concerns about radiation. Moreover, the measurement of obesity based on CT doesn't seem much better than WC measurement in patients with subclinical coronary heart disease (19). In contrast, anthropometry measurements, like WC and WHR, which have been proven to be related to visceral fat, are easy to perform at a low cost. Thus, anthropometry measurements are widely used to measure body fat distribution and widely applied in the clinic (20, 21). Although there is no significant difference between WHR, WC, and WHtR in terms of their influence on clinical outcomes (22), WHR is considered more accurate to define abdominal obesity than WC for patients with large body size. Individuals with large body size without abdominal obesity may be misdiagnosed as having abdominal obesity because of the high WC (23). WHR is demonstrated to be related to the risk of CHD (24), therefore, we used WHR as a measurement of abdominal obesity in this study.

Although cardiopulmonary function is closely related to the prognosis of HF, it is not well-utilized in the clinical practice due to measuring difficulty and lack of standard (25). As an essential measurement of cardiopulmonary function, cardiopulmonary exercise test (CPET) and its indices are recognized as a certain influencing factor of the prognosis of HF (26–28). With the above background, this study aimed to assess the influence of WHR on the prognosis of HF patients with revascularized CHD.

## Materials and Methods

### Study Population

Consecutive patients who were referred to the Cardiac Rehabilitation Clinic of PLA Hospital from June 30, 2015, to June 30, 2019, were invited in our study. The Ethics Committee of PLA General Hospital approved the study and all participants provided written informed consent (registration number: ChiCTR2000035048). This study is a prospective study.

The inclusion criteria were as follows: (1) age between 18 and 80 years, (2) diagnosis of CHD [in accordance with the 2012 ACCF/AHA focused update of the guideline for the management of patients with unstable angina/Non-ST-elevation myocardial infarction (29)] and underwent revascularization, (3) diagnosis of HF [in accordance with 2013 ACCF/AHA guideline for the management of heart failure (30)], (4) available WHR data and CPET results, and (5) left ventricular ejection fraction (LVEF) lower than 50%. The exclusion criteria were as follows: (1) HF due to non-ischemic cardiomyopathy (such as dilated cardiomyopathy and hypertrophic cardiomyopathy), (2) severe angina, (3) uncontrolled arrhythmia, and (4) untreatable carcinoma. Baseline information including demographic characteristics (such as age, sex, etc.), clinical features (such as diagnosis, history of the disease, etc.), complications (such as hypertension, diabetes, etc.), medicine, CPET results, cardiac ultrasound results, and laboratory results were collected 3 months before and after CPET from the database of the Cardiac Rehabilitation Clinic of PLA hospital.

### Waist and Hip Circumference Measurement

Trained nurses used uniform standards during measurement. The waist and hip circumference were measured when patients were standing, wearing light clothing. At the end of expiration and the beginning of inspiration, WC was measured at the midpoint between the lowest point of the rib and the upper edge of the iliac crest. Hip circumference was measured at the most prominent part of the buttocks. The measurement of WC and hip circumference had an accuracy of 0.1 cm. The average WC and hip circumference were calculated from three measurements. WC divided by hip circumference was defined as WHR.

### CPETs

Every patient enrolled in our study performed the cardiopulmonary exercise test using the stationary cycle ergometer and gas analysis apparatus (CS-200, Schiller, Obfelden, Switzerland). The breath-by-breath method was used to analyze gas exchanges. Mixed gases (4%CO_2_/16%O_2_/N_2_) were used for calibration before each test, and the test was performed using the ramp protocol. The exercise duration was 8–12 min. The ramp protocol was carried out as follows: the patient rested for 1 min with a load of 0 W, performed warm-up exercises for 2 min with a load of 0 W, and continued the exercise with an initial load of 5 W. The load was further increased in the ramp-incremental exercise (25 W/min in men, 20 W/min in women). The speed ranged from 55 to 65 rpm until the maximal load. For the recovery protocol, the patient performed exercise with load of 0 W for at least 2 min. When ST depression was ≥3 mm, the systolic blood pressure or average blood pressure decreased by ≥10 mmHg, angina or severe arrhythmia occurred, or the patient requested to stop the exercise, the exercise load was removed and the test was stopped.

### Outcomes

The primary outcome in this study was the occurrence of major adverse cardiac events (MACE), including all-cause mortality, non-fatal myocardial infarction, unscheduled revascularization, and stroke. All-cause mortality was defined as death from any cause. Non-fatal myocardial infarction was defined according to ESC guidelines (31). Unscheduled revascularization was defined as balloon dilatation, percutaneous coronary intervention, or coronary artery bypass grafting surgery unexpectedly. Stroke was defined as ischemic or hemorrhagic nervous system disease which is not secondary to brain tumors, brain trauma, or other reasons. Clinical end-point events were determined by the steering committee.

### Follow-Up

Clinical outcomes were collected from clinic visits, 6-month telephone interviews, or medical history from our hospital's database. We contacted the patients or their families by phone call prior to recording their outcomes.

### Statistical Analysis

Normal data are presented as mean ± standard deviation, and non-normal data are presented as median (25th percentile, 75th percentile). Cox proportional hazards models were used to determine whether WHR was independently associated with MACE. Patients were divided into two groups according to the WHR cutoff value calculated by the X-tile software. Kaplan–Meier analysis and log-rank test were used to assess significant differences in survival time and survival differences between the two groups. Hazard ratio (HR) and confidence interval (CI) between the higher and lower WHR groups were calculated by Cox proportional risk regression. Further adjustments included possible predictors of abdominal obesity, HF, or coronary heart disease. We computed receiver operating characteristic (ROC) curves to evaluate the value of WHR in predicting MACE incidence within 1, 2, 3, and 4 years. The characteristics of the two groups were also compared. Chi-square test, Fisher's exact test, or Mann–Whitney *U*-test was used to compare categorical variables, while Student's *t*-test or Mann–Whitney *U*-test was used to compare continuous variables, as appropriate. A *P* < 0.05 was considered statistically significant. Statistical analyses were performed using IBM SPSS Statistics version 19 and R Language Version 3.6.3.

## Results

### General Characteristics of the Study Participants

**Figure 1:**
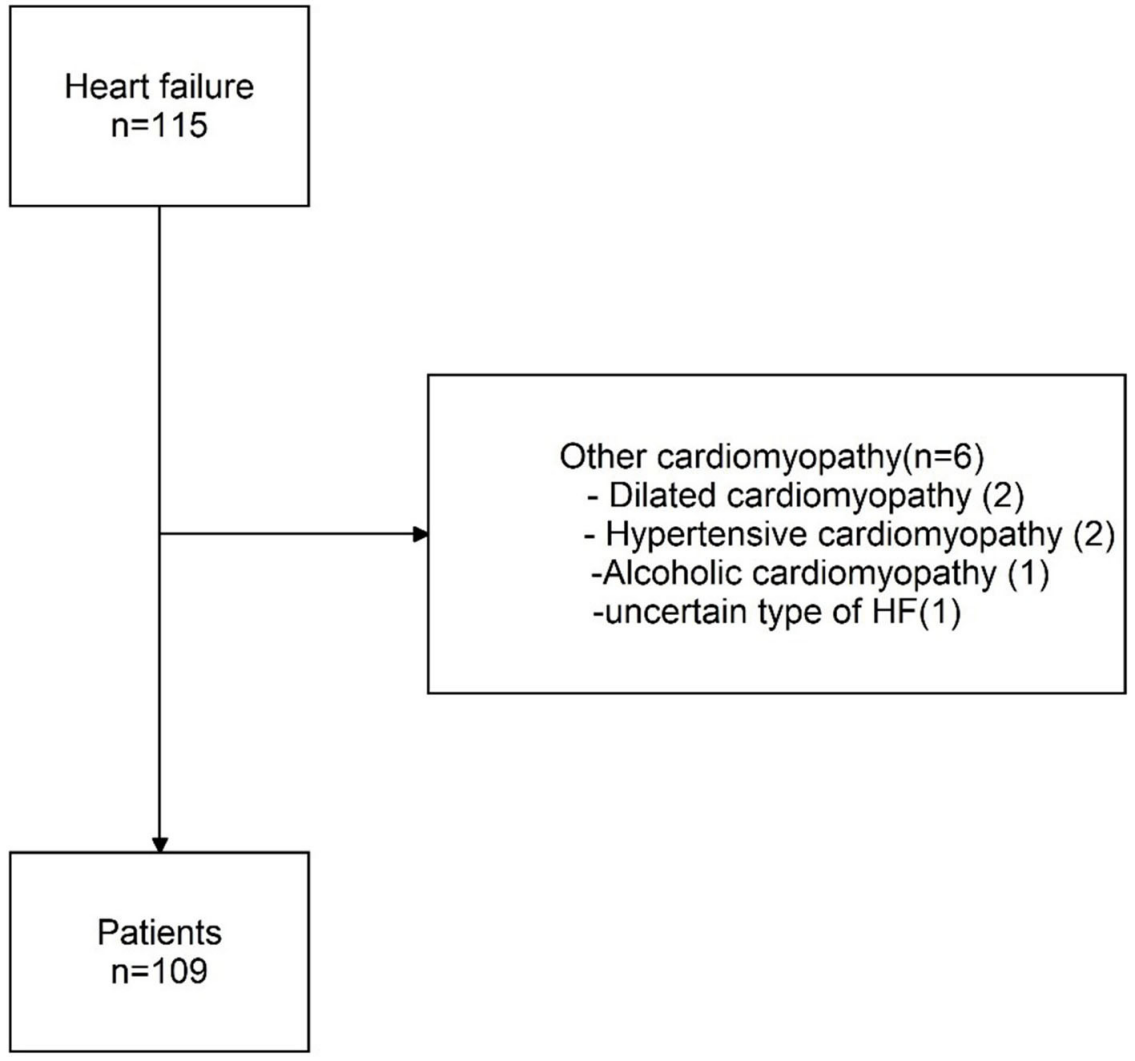
Study cohort. HF, heart failure; CHD, coronary heart disease.

Of the 115 patients, 109 were enrolled in our study, and the reasons of exclusion are shown in Figure 1. The majority [101 (91.7%)] of the patients were men, with a mean age of 56 ±10 years. The patients tended to be obese with higher WHR [0.93 (0.90–0.96)] and higher BMI (26.1 ± 3.1) kg/m_2_. Patients were more often at New York Heart Association I [55 (50.5%)] and were more often classified as having HF with mid-range ejection fraction [92 (84.4%)]. Cardiac ultrasonography showed a median LVEF of 44.5% (5). Most of the patients [81 (79.4%)] had smoking history. The majority of the patients [99 (90.8%)] were diagnosed of old myocardial infarction. Common complications were diabetes [32 (29.4%)], hypertension [59 (54.1%)], and hyperlipidemia [56 (52.3%)]. Nearly all patients took antiplatelet drugs [98 (97.0%)] and beta-blockers [81 (80.2%)] (Table 1).

**Table 1:** Baseline characteristics of study participants.

**Characteristic**	**Mean ± std/median (25–75 p)/No of cases (%)**
**Demographic characteristics**	
Sex, male	100 (91.7)
Age, years	56.0 ± 10.4
WHR	0.93 (0.90–0.96)
BMI, kg/m^2^	26.1 ± 3.1
**BMI grade**	
<18.5 kg/m^2^	0 (0)
18.5–24.9 kg/m^2^	37 (33.94)
25.0–29.9 kg/m^2^	60 (55.05)
≥30.0 kg/m^2^	12 (11.01)
**NYHA class**	
I	55 (50.5)
II	39 (35.8)
III	8 (7.3)
IV	7 (6.4)
**LVEF (%)**	
HFrEF	17 (15.6)
HFmrEF	92 (84.4)
SBP	125.7 ± 14.6
DBP	80.2 ± 10.4
LVEF, %	44.5 (41–46)
Smoking history	81 (79.4)
**Medical history**	
MI	99 (90.8)
Hypertension	59 (54.1)
Diabetes	32 (29.4)
Hyperlipidemia (*n* = 107)	56 (52.3)
**Pharmacotherapy (*****n*** **=** **101)**	
ACEI	31 (30.7)
ARB	11 (10.9)
Beta-blocker	81 (80.2)
Statins	96 (95.1)
Diuretic	17 (16.8)
Antiplatelet agents	98 (97.0)
Digoxin	5 (5.0)

*WHR, waist-to- hip ratio; BMI, body mass index; NYHA, New York Heart Association; LVEF, left ventricular ejection function; HFrEF, heart failure with reduced ejection fraction; HFmrEF, heart failure with mid-ranged ejection fraction; SBP, systolic blood pressure; DBP, diastolic blood pressure; MI, myocardiopathy infraction; ACEI, angiotensin converting enzyme inhibitors; ARB, angiotensin receptor blocker*.

### Outcomes

The median survival time was 39 months (interquartile range, 14), and the maximum survival time was 54 months. During the follow-up, 3 (2.8%) patients died due to cardiac-related death (*n* = 2) or gastrointestinal hemorrhage (*n* = 1); 1 (0.9%) patient had a non-fatal myocardial infarction, and 13 (11.9%) patients had unscheduled revascularization (Table 2).

**Table 2:** Outcomes.

**Outcomes**	**No of cases (%)**
MACE	16 (14.7)
Death	3 (2.8)
Cardiac death	2 (1.8)
Non-cardiac death	1 (0.9)
Unscheduled revascularization	13 (11.9)
Non-fatal myocardial infarction	1 (0.9)
Stroke	0 (0)

*MACE, major adverse cardiac events*.

### Association of WHR With Unfavorable Outcomes of HF Patients With Revascularized CHD

Cox analysis results demonstrated WHR might be an independent predictor of the incidence of MACE (*P* < 0.001); i.e., an increase of 0.01 unit in WHR correspond to a HR of 1.134 (95%CI: 1.057–1.216). We divided the patients into two groups according to WHR cutoff value (0.93) calculated by X-tile. The incidence of MACE differed between the higher and lower WHR groups: there was a significant difference in the incidence of MACE (23.6 vs. 5.6%, *P* = 0.008) and in the incidence of unscheduled revascularization (20.0 vs. 3.7%, *P* = 0.015). No statistically significant difference was noted in the all-cause mortality or incidence of non-fatal myocardial infraction between the two groups (*P* > 0.05; Table 3).

**Table 3:** Outcomes in groups stratified by WHR.

	**WHR < 0.93** **(*n* = 54)**	**WHR ≥ 0.93** **(*n* = 55)**	***P***
MACE	3 (5.6)	13 (23.6)	0.008
Death	1 (1.9)	2 (3.6)	1
Cardiac death	0 (0)	2 (3.6)	0.495
Non cardiac death	1 (1.9)	0 (0)	0.495
Unscheduled revascularization	2 (3.7)	11 (20.0)	0.015
Non-fatal myocardial infarction	0 (0)	1 (1.8)	1

*Data are expressed as No of cases (%). MACE, major adverse cardiac events*.

**Figure 2:**
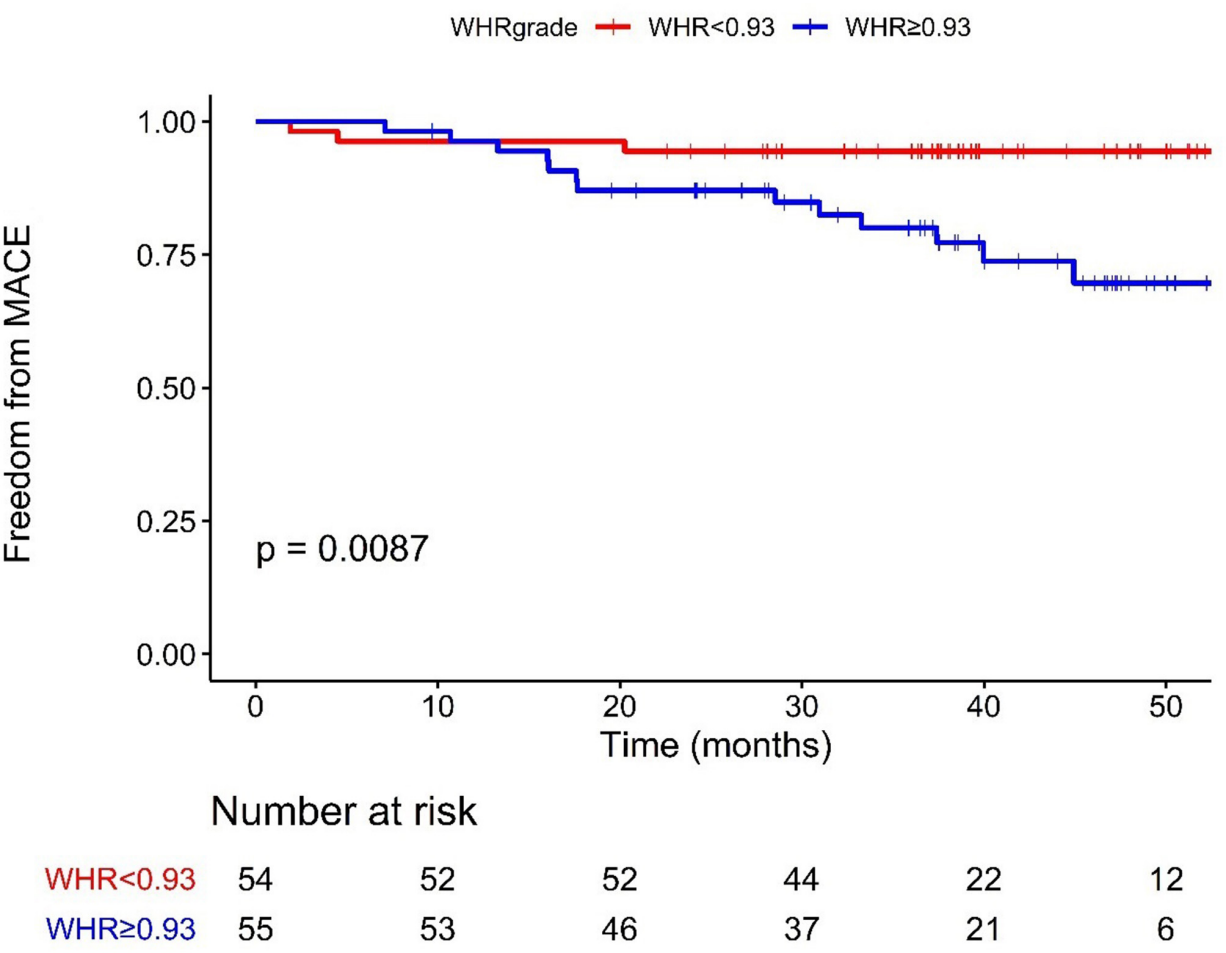
Kaplan–Meier curves of MACE stratified according to the cut-off value. WHR < 0.93 (54 cases), ≥0.93 (55 cases); MACE, major adverse cardiac events; WHR, waist-to-hip ratio.

Figure 2 presents the Kaplan–Meier survival curves between the higher and lower WHR groups. Patients in the lower WHR group presented much higher event-free survival possibility than patients in the higher WHR group [*P* = 0.0087]. In the univariate analysis, patients in the higher WHR group were more likely to experience MACE (HR = 4.611, 95%CI: 1.313–16.193). After adjustment for age, sex, BMI, diabetes, hypertension, HR rest as well as CPET parameters, including VO2_kg_AT, VO2_max, VE/VCO2_slope, patients in the higher WHR group were still more likely to experience MACE (HR = 7.037, 95%CI: 1.758–28.168; Table 4).

**Table 4:** Hazard ratio and *P*-value of higher WHR group vs. lower WHR group and MACE.

	**HR (95%CI)**	***P***
Crude	4.611 (1.313–16.193)	0.017
Model 1	4.921 (1.391–17.406)	0.013
Model 2	5.487 (1.507–19.973)	0.01
Model 3	7.037 (1.758–28.168)	0.006

*Model 1 was adjusted for age and sex*.

*Model 2 further adjusted for age, sex, BMI, diabetes, hypertension, and HR rest*.

*Model 3 further adjusted for age, sex, BMI, diabetes, hypertension, HR rest, VO2_kg_AT, VO2_max, VE/VCO2_slope*.

*WHR, waist-to- hip ratio; BMI, body mass index; LVEF, left ventricular ejection function; HR, heart rate; SBP, systolic blood pressure; ACEI, angiotensin converting enzyme inhibitors; ARB, angiotensin receptor blocker*.

**Figure 3:**
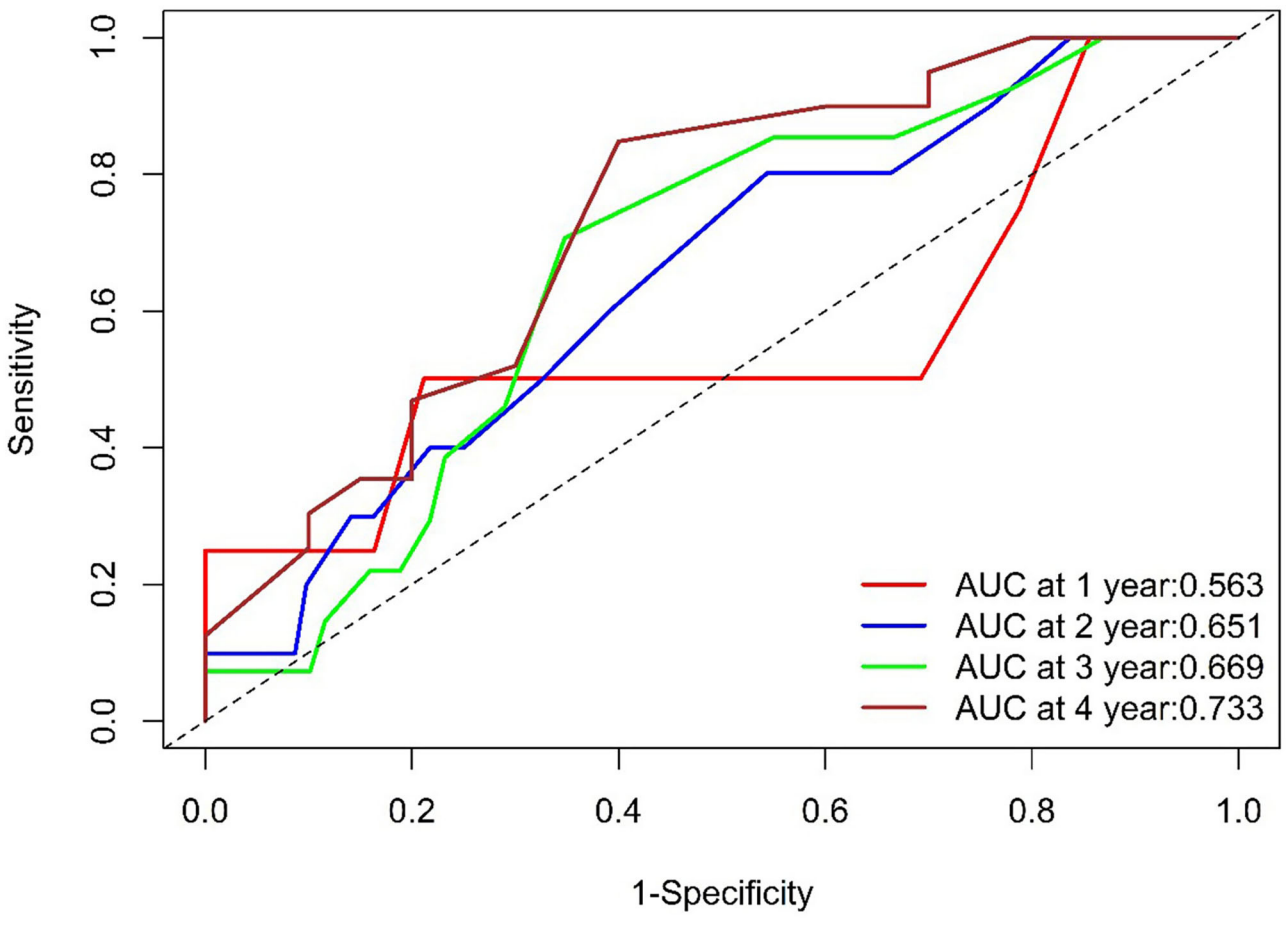
ROC of WHR. The WHR AUC was 0.563 at 1 year (104 survivors and 4 events), 0.651at 2 years (92 survivors and10 events), 0.669 at 3 years (69 survivors and 13 events), and 0.733at 4 years (20 survivors and 16 events). ROC, Receiver Operating Characteristic; WHR, waist-to- hip ratio; AUC, area under the curve.

The ROC curves for WHR and MACE are displayed in Figure 3. The ROC area under curve (AUC) was 0.563 at 1 year, 0.651 at 2 years, 0.669 at 3 years, and 0.733 at 4 years.

The descriptions of patients' characteristics are shown in Table 5, dichotomized by the WHR cutoff value. Patients with higher WHR had a higher level of BMI (26.7 ± 3.5 vs. 25.4 ± 2.4, *P* = 0.033) and ΔVO2/ΔWR [10.2 (8.7–13.5) vs. 12.0 (9.9–14.0), *P* = 0.025] than those with lower WHR. No significant difference was found between the two groups in terms of age, sex, cardiac function classification, blood pressure, smoking history, medical history, medication, cardiac ultrasound results and other CPET parameters (*P* < 0.05).

**Table 5:** Comparison of clinical characteristic between groups stratified by WHR.

**Characteristic**	**WHR < 0.93**	**WHR ≥ 0.93**	***P***
Male, *n* (%)	50 (92.6)	50 (90.9)	1
Age, years	57.5 ± 10.0	54.7 ± 10.6	0.16
BMI, kg/m^2^	25.4 ± 2.4	26.7 ± 3.5	0.033
**NYHA class**			0.821
I	29 (53.7)	26 (47.3)	
II	16 (29.6)	23 (41.8)	
III	4 (7.4)	4 (7.3)	
IV	5 (9.3)	2 (3.6)	
**LVEF (%)**			0.201
HFrEF	6 (11.1)	11 (20)	
HFmrEF	48 (88.9)	44 (80)	
SBP, mmHg	126.1 ± 14.8	125.2 ± 14.5	0.739
DBP, mmHg	79.5 ± 9.7	80.9 ± 11.2	0.503
LVEF, %	44 (41–46)	45 (40–46)	0.732
Smoking history	41 (78.9)	40 (80)	0.885
**Medical history**, ***n*** **(%)**			
Hypertension	31 (57.4)	28 (50.9)	0.496
Diabetes	17 (31.5)	15 (27.3)	0.630
Hyperlipidemia	28 (52.8)	28 (51.9)	0.995
**Pharmacotherapy**, ***n*** **(%)**			
ACEI	14 (26.9)	17 (34.7)	0.397
ARB	6 (11.5)	5 (10.2)	0.507
Statins	48 (92.3)	48 (98.0)	0.363
Beta-blocker	44 (84.6)	37 (75.5)	0.251
Diuretic	8 (15.4)	9 (18.4)	0.689
Antiplatelet	50 (96.2)	48 (98.0)	1
**CPET**			
Peak VO2, ml/ (kg·min)	16.3 ± 0.6	17.3 ± 0.6	0.246
VO2 at AT, ml/ (kg·min)	12.3 (10.0–14.2)	12.3 (10.0–14.1)	0.058
O2 pulse, ml/bpm	9.8 ± 0.4	11.0 (8.9–12.2)	0.110
VE/VCO_2_ slope	26.5 (23.1–29.6)	28.2 ± 0.6	0.063
ΔVO2/ΔWR, ml/ (min·W)	10.2 (8.7–13.5)	12.0 (9.9–14.0)	0.025

*Data are expressed as mean ± std, median (25–75 p), No of cases (%)*.

*WHR, waist-to- hip ratio; BMI, body mass index; NYHA, New York Heart Association; LVEF, left ventricular ejection function; HFrEF, heart failure with reduced ejection fraction; HFmrEF, heart failure with mid-ranged ejection fraction; SBP, systolic blood pressure; DBP, diastolic blood pressure; ACEI, angiotensin converting enzyme inhibitors; ARB, angiotensin receptor blocker*.

## Discussion

Abdominal obesity may be related to the prognosis of CHD, while the value is not sure in HF patients, especially in HF patients with revascularized CHD. In this study, by collecting the data of 109 revascularized CHD patients with HF with a maximum follow-up time of 54 months, we for the first time found WHR was associated with the incidence of MACE in Chinese HF patients with revascularized CHD (*P* < 0.001). Every 0.01 increase in WHR had a corresponding ~13.4% higher risk to develop MACE. Patients in the higher WHR group had a higher risk of MACE than patients in the lower WHR group. The HR increased to 7.037 after adjustment for multivariables (Table 4). The ROC AUC was 0.733 at 4 years. Thus, our main finding was that WHR might be an independent risk factor of the long-term prognosis. Additionally, BMI level and ΔVO2/ΔWR in the higher WHR group demonstrated higher than that in the lower WHR group.

Abdominal obesity is associated with the high mortality in CHD (32) and is a risk factor of the prognosis of CHD. Although the prognosis of CHD patients improved by the development of optimal medical therapy and revascularization technology, the incidence of HF due to myocardial infarction was still high (33), and the risk of death for HF patients due to myocardial infarction increased to 3–4-folds, compared with myocardial infarction patients without HF (34). Patients with HF due to CHD have poor prognosis. Thus, there is a need to find predictors to improve the prognosis of these patients.

The all-cause mortality of our cohort was 2.8% (Table 2). In this study, the median LVEF was 44.5 (35–40) %, which may be one possible reason of the low mortality. Furthermore, although 90% of the patients in this study had HF due to myocardial infarction, all patients were successfully revascularized. In addition, 97.0% of the patients received antiplatelet agents and 80.2% took beta-blockers. Most patients have received optimal medical therapy which might attribute to the low mortality.

Further Cox regression analysis showed WHR independently related to the incidence of MACE for HF patients with revascularized CHD (P <0.001); an increase of WHR by 0.01 unit correspond to ~13.4% higher risk. To better guide clinical practice, we divided the patients into two groups stratified by WHR cutoff value of 0.93. Patients in the higher WHR group had a significantly higher risk of MACE than patients in the lower WHR group. After multivariable adjustment, the higher WHR remained significantly associated with a higher incidence of MACE. Kaplan–Meier and log-rank test analysis showed similar tendency.

Our findings were consistent, to a certain extent, with the findings of some studies reporting that WHR was associated with rehospitalization due to HF (41). WHR might affect the prognosis of HF patients with revascularized CHD in the following mechanism: WHR increased in patients with abdominal obesity, which is an external manifestation of visceral fat accumulation. To our knowledge, the accumulation of visceral adipose tissue regulates sympathetic hyperactivity through the direct influence of the autonomic nervous system. Meanwhile, the increase in visceral fat will change the secretion mode of adipocytokines (including leptin, adiponectin, etc.), which plays a vital role in insulin resistance, dyslipidemia, prethrombotic state, and chronic inflammatory state. These abnormal clinical conditions will eventually promote the development of coronary atherosclerosis and adverse events of HF (15, 42–46). Besides, abdominal obesity is associated with LV longitudinal strain and increased epicardial adiposity, which also increases the incidence of adverse cardiovascular effects (35, 36). Higher WHR may influence the prognosis of HF patients with revascularized CHD directly or indirectly through the above mechanisms.

We observed that the AUC was 0.563, 0.651, 0.669, and 0.733 (>0.7) at 1, 2, 3, and 4 years, respectively (Figure 3), which meant that WHR had more prognostic value in the long-term in HF patients with revascularized CHD. However, further studies with large sample are required to validate the above finding.

In this study, we also compared the differences in age, sex, cardiac function, and complications between the higher and the lower WHR groups. The difference in BMI was statistically significant. Streng et al. (23) found that the increase in WHR was associated with an increase in weight, which led to an increase in BMI. Ortega et al. (37) thought that BMI in abdominal obesity patients was higher than patients without abdominal obesity. The relationship between BMI and abdominal obesity still existed in HF patients with revascularized CHD, which may be related to common risk factors, including unhealthy diet patterns, low physical activities, and low cardiorespiratory fitness. ΔVO2/ΔWR, as a sensitive indicator of abnormal muscle oxygen transport or utilization during exercise, was higher in the higher WHR group, which may be attributed to the increased functional impairment and higher cardiopulmonary stress (38, 39). In addition, no significant differences were observed in other indicators including age, sex, cardiac function classification, medical history, and medication.

WHR was focused in Chinese revascularized HF patients for the first time. The World Health Organization recommended WHR ≥ 0.9 for men and WHR ≥ 0.85 for women as the standard diagnostic of abdominal obesity. In our study, we determined 0.93 as the cutoff value by calculation using X-tile software, which is extensively used (40). Consequently, we found a significant difference in the prognosis between patients with higher and lower WHR. Thus, patients with a WHR ≥ 0.93 in Chinese HF patients with revascularized CHD population, deserve more attention and intensified treatment. The meaning of abdominal obesity deserves further research in revascularized HF patients.

This study has some limitations. First, this study had a small sample size and involved a single center, although the follow-up duration was long. Thus, a multicenter study with large size is under consideration. Second, sex and ethnicity might influence fat distribution (47). The WHR cutoff value (0.93) in our study did not distinguish between male and female patients, and it was only aimed at the Chinese population. Further study with larger size and more stratification might resulted in more accurate WHR cutoff value. Incomplete revascularization may affect MACE, while the information of incomplete revascularization was not collected.

## Conclusion

In conclusion, this study shows that WHR demonstrated to be an independent risk factor of the long-term prognosis of Chinese HF patients with revascularized CHD. Patients with higher WHR are more vulnerable to develop MACE in the long term. Thus, Patients with WHR ≥ 0.93 need more concern and require intensified treatment. Higher WHR is related to higher BMI and higher ΔVO2/ΔWR, which may be associated with the common risk factors of abdominal obesity and obesity.

## Data Availability Statement

The raw data supporting the conclusions of this article will be made available by the authors, without undue reservation.

## Ethics Statement

The studies involving human participants were reviewed and approved by Ethics Committee of PLA General Hospital. The patients/participants provided their written informed consent to participate in this study.

## Author Contributions

JM designed and helped in the data analysis and manuscript writing. YX and YC contributed to the conception of the study. YiZ and YaZ performed the data analyses and wrote the manuscript. YS performed the CPET and collected the data. WD, YM, JW, YG, and RH referred the participants and helped perform the analysis with constructive discussions. All authors contributed to the article and approved the submitted version.

## Funding

This work was supported by the National Key R&D Program of China (2018YFC2000600).

## Conflict of Interest

The authors declare that the research was conducted in the absence of any commercial or financial relationships that could be construed as a potential conflict of interest.

## Publisher's Note

All claims expressed in this article are solely those of the authors and do not necessarily represent those of their affiliated organizations, or those of the publisher, the editors and the reviewers. Any product that may be evaluated in this article, or claim that may be made by its manufacturer, is not guaranteed or endorsed by the publisher.
